# Pathological Change and Whole Transcriptome Alternation Caused by ePTFE Implantation in Myocardium

**DOI:** 10.1155/2021/5551207

**Published:** 2021-06-18

**Authors:** Yaping Shan, Wenbo Zhang, Gang Chen, Qiqi Shi, Yaping Mi, Huifeng Zhang, Bing Jia

**Affiliations:** Department of Cardiovascular Center, Children's Hospital of Fudan University, Shanghai 201102, China

## Abstract

Expanded polytetrafluoroethylene (ePTFE) is commonly used in cardiovascular surgery, but usually causes postoperation complications. Although great efforts have been done to relieve these complications or to understand their mechanism, there are no applicable strategies available and no understanding mechanisms, especially in the myocardium. Here, ePTFE membranes are implanted into the right ventricular outflow tract of rabbits, and the implant-related myocardium is dissected and analyzed by histology and transcriptome sequencing. ePTFE implantation causes myocardium inflammation and fibrosis. There are 1867 differently expressed mRNAs (DEmRNAs, 1107 upregulated and 760 downregulated) and 246 differently expressed lncRNAs (DElncRNAs, 110 upregulated and 136 downregulated) identified. Bioinformatic analysis indicates that the upregulated DEmRNAs and DElncRNAs are mainly involved in inflammatory, immune responses, and extracellular matrix remodeling, while the downregulated DEmRNAs and DElncRNAs are predominantly functioned in the metabolism and cardiac remodeling. Analysis of coexpression and regulatory relationship of DEmRNAs and DElncRNAs reveals that most DElncRNAs are *trans*-regulated on the relevant DEmRNAs. In conclusion, ePTFE implantation causes severe myocardial tissue damages and alters the transcriptome profiles of the myocardium. Such novel data may provide a landscape of mechanisms underlying the adverse reactions caused by ePTFE implantation and uncover new therapeutic targets for inhibiting the ePTFE-related complications.

## 1. Introduction

Expanded polytetrafluoroethylene (ePTFE) is a durable high polymer material and has been widely used in cardiovascular surgery, such as bypass grafting [1], right ventricular outflow tract reconstruction [2], the Blalock-Taussig (BT) shunt [3], and valve replacement [4]. However, ePTFE naturally is a foreign material to human recipients and may induce a foreign body reaction (FBR), which impairs the function of ePTFE and human organs.

A previous study has pointed that ePTFE grafts can cause vascular stenosis, particularly for a graft at <6 mm in diameter, even in the use of anticoagulants [5]. As a result, the 2-year failure rate of ePTFE graft in dialysis access sites is 75% [6], the 5-year stenosis rate of ePTFE graft in femoropopliteal bypass ranges between 50% and 75% [7], and the BT shunt-related stenosis often causes acute death in children [3]. Furthermore, the long-term use of anticoagulants is usually associated with side effects, such as bleeding. Currently, the ePTFE graft-related stenosis is mainly attributed to intimal hyperplasia, thrombosis, and inflammation [6, 8–10]. Accordingly, many efforts have tried to improve the anticoagulant properties of ePTFE and they include heparin coating [1], endothelial cell coverage [6], antibody or peptide coating the ePTFE [11–13], and others [14, 15]. Similarly, several methods have been developed to prevent intimal hyperplasia or inhibit the FBR [16–19]. However, their efficacy and stability are unsatisfactory and far away from clinical application [20, 21]. The pathogenesis of ePTFE graft failure remains unclear.

While ePTFE implants are necessary for reestablishment of effective blood flow in some pathological conditions, such as children with a complex congenital heart disease, the ePTFE implants can not only damage the blood vessel but also damage the myocardium. Given that the myocardium has little potential to regenerate, the myocardial damages caused by an ePTFE implant are usually irreversible, leading to impaired heart function and even to death. Previous studies have revealed the pathogenesis of ePTFE graft-related vascular injury [22–25]. However, little is known on the pathological changes in the myocardial tissues and molecular mechanisms underlying the pathogenesis of ePTFE graft-related myocardial damages.

This study employed a rabbit model of ePTFE implant-related myocardial injury and used bioinformatics to analyze the relative levels of mRNA and long noncoding RNA (lncRNA) transcripts in the implant-related myocardial tissues to explore the potential molecular pathogenesis.

## 2. Materials and Methods

### 2.1. Ethics Statement

The experimental protocol was approved by the Animal Care and Ethical Committee of Children's Hospital of Fudan University, and animal experiments were performed, according to the guidelines of the Animal Care Committee.

### 2.2. Animal Model and Groups

Female New Zealand rabbits at 3 months of age and weighing 2.5~3.5 kg were obtained from Shanghai Jiagan Biological Science and Technology, China. All rabbits were housed and raised in a normal environment. Some rabbits served as the control group without operation. The remaining rabbits were food-fasted for 6 hours, anesthetized intramuscularly with 15 mg/kg of Zoletil® 50 (Virbac S.A., France), and oxygenized with a mask. The rabbits were fixed on an operation table, and the sternum was cut in median to expose the right ventricular outflow tract of the heart. The rabbits were implanted with an ePTFE membrane (1 × 0.4 cm, Gore, USA) and they were defined as the ePTFE group, and the remaining rabbits received sham surgery (sham group). Briefly, the right ventricular outflow tract of individual rabbits was cut and inserted with the ePTFE membrane into the right ventricle, followed by suturing its one tail onto the myocardium with 5-0 Prolene. The rabbits in the sham group received similar surgery without inserting the membrane.

### 2.3. Tissue Collection, Pathologic Analysis, and RNA Extraction

On day 30 postsurgery, all rabbits were anesthetized, and their heart tissues were dissected out. Their myocardial tissues linked to the ePTFE membrane (ePTFE group) or linked to the sutured parts (sham group) or similar locational tissues in the control group (control group) were dissected on ice.

One part of the myocardial tissues was fixed with 4% formaldehyde solution for 48 h and paraffin embedded. The tissue sections (4 *μ*m) were stained with hematoxylin and eosin (H&E) or Masson's trichrome. The pathological changes in individual sections were examined under a light microscope, and the degrees of fibrosis were calculated using HALO software (Indica Labs, USA).

The remaining myocardial tissues were stored in RNA later (Invitrogen, USA) at -80°C. Total RNA was extracted from individual myocardial samples using the mirVana miRNA Isolation Kit (Ambion, USA), following the manufacturer's protocol. The quality and quantity were evaluated using the Agilent 2100 Bioanalyzer (Agilent Technologies, Santa Clara, CA, USA). The samples with a RNA integrity number (RIN) ≥ 7 were used for the subsequent analysis.

### 2.4. Construction of cDNA Libraries and High-Throughput Sequencing

Strand-specific libraries were prepared using the TruSeq Stranded Total RNA with Ribo-Zero Gold (Illumina, USA). Briefly, after removal of ribosomal RNA, the fragmented RNA was reversely transcribed into cDNA. The cDNA was purified and individual DNA fragments were added with adenylate at the 3′end, followed by ligating adapters. The cDNA fragments were further purified and enriched with PCR to create the final cDNA libraries, which were analyzed by the Agilent 2100 Bioanalyzer (Agilent Technologies, USA) to confirm the insert sizes and calculate the concentrations. The libraries were sequenced on an Illumina HiSeq X Ten platform at OE Biotech (Shanghai, China) to generate 150 bp/125 bp paired-end reads.

The generated raw reads were processed by removing the adapter, low-quality of bases and N-bases using Trimmomatic software [26]. Finally, the remaining high-quality clean reads were aligned to the *Oryctolagus cuniculus* reference genome (OryCun 2.0, NCBI) using hisat2 [27]. The aligned data were stored in a binary file, called a bam file, and were assembled into new transcripts using the StringTie software. Subsequently, the candidate lncRNA transcripts were selected by comparing the gene annotation information of the reference sequence produced by the Cuffcompare software. Finally, transcripts with coding potential were analyzed using the CPC, CNCI, Pfam, and PLEK to obtain lncRNA-predicted sequences.

### 2.5. Quantification of Gene Expression and Differential Expression Analysis

The expression levels of individual genes were estimated by fragments per kilobase of exon model per million reads mapped (FPKM). After standardized gene counts from every group, the fold change (FC) and significant difference between groups were calculated using the Negative Binomial (NB) distribution test. The differentially expressed mRNAs (DEmRNAs) and differentially expressed lncRNAs (DElncRNAs) were determined when *P* < 0.05 and FC > 2. The distribution of these DEmRNAs and DElncRNAs was analyzed by volcanic plots. The location and expression levels of all identified DEmRNAs and DElncRNAs on chromosomes between different groups were analyzed by a Circos plot. The similarity of DEmRNAs and DElncRNAs between the groups was analyzed by Venn analysis. The expression pattern of DEmRNAs and DElncRNAs in all samples was illustrated by heatmaps.

### 2.6. Quantitative RT-PCR and Validation

The RNA sequence data were validated by quantitative RT-PCR. A total of 6 (3 upregulated, 3 downregulated) DEmRNAs and 6 (3 upregulated, 3 downregulated) DElncRNAs were chosen, respectively. The relative levels of these RNA transcripts were determined by a two-step quantitative RT-PCR on a GeneAmp® PCR System 9700 (Applied Biosystems). These RNA samples were reversely transcribed into cDNA using the HiScript II Q RT SuperMix (Nanjing, China). The relative levels of the gene transcripts to the control GAPDH were quantified by PCR using ChamQ SYBR qPCR Master Mix (Nanjing, China) and specific primers (Generay Biotech, Shanghai, China, Table 1). The data were analyzed by the 2^-*ΔΔ*Ct^ method and were transformed into FC using the following formula:

(1) $$\begin{array}{c} \text{FC}=\mathrm{lg}\left(\frac{\text{RNA expression level in the ePTFE or sham group}}{\text{RNA expression level in the control group}}\right). \end{array}$$

### 2.7. Functional Enrichment Analysis

The function of DEmRNAs in the ePTFE vs. control comparison was predicted by using Gene Ontology (GO) and Kyoto Encyclopedia of Genes and Genomes (KEGG) enrichment analysis. GO terms comprised three function domains: biological process (BP), cellular component (CC), and molecular function (MF). The 6 items with the smallest *P* values or all items if fewer than 6 met the statistical criteria in each GO term were selected for graphical display. The distribution of whole DEmRNAs in KEGG level 2 was illustrated, and the 20 items with the smallest *P* values were selected for bubble diagram display or all the items if fewer than 20 met the statistical criteria. A signal pathway map was constructed to show the relationship of different pathways.

### 2.8. Coexpression Analysis of DElncRNAs and DEmRNAs

Because the functions of most lncRNAs have not been identified, we analyzed the coexpression of DElncRNAs and DEmRNAs using the Pearson correlation coefficient (PCC) with the cut-off values of ∣PCC | >0.8 and *P* < 0.05. The top 10 DElncRNAs were further analyzed because they had coexpressed mRNAs in the most GO terms or KEGG pathways, and these mRNAs were enriched by ≥5. The function of these DElncRNAs was predicted by GO and KEGG enrichment analysis, based on their coexpressed DEmRNAs.

### 2.9. *Cis*- and *trans*-Regulation of Predicted DElncRNAs

An lncRNA regulated the transcription of mRNA within the 100 kb both its upstream and downstream on the same chromosome, which was defined as *cis*-regulation relationship. The *trans*-regulation relationship was defined when the coexpressed lncRNA and mRNA on different chromosomes were chosen and their binding was based on >10 bases with the free energy of bases binding ≤−50, analyzed using RNA interaction software RIsearch-2.0. We selected the DEmRNAs between the ePTFE and healthy controls into (1) GO term related to inflammatory response, immune response, and complex binding and (2) GO term related to smooth muscle cell and extracellular matrix. Subsequently, we selected DElncRNAs with *cis*- or *trans*-relationship with those selected DEmRNAs to construct the regulation network for the potential regulation mechanisms.

### 2.10. Statistical Analysis

The statistical analysis methods for RNA sequencing data were described above. The difference in the fibrosis degrees between groups was analyzed by Student's *t*-test. A *P* value of <0.05 was regarded as statistically significant. All statistical analyses were performed using the SPSS 19.0 software (IBM SPSS Statistics, USA), and the graphs were made using the GraphPad Prism 8 Software (GraphPad, USA).

## 3. Results

### 3.1. ePTFE Implantation Induces Myocardial Tissue Injury

**Figure 1:**
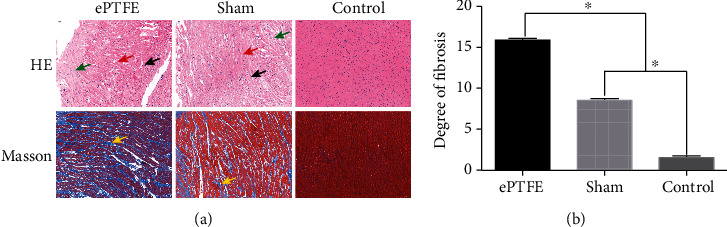
ePTFE implantation induces myocardial tissue injury. (a) HE staining displayed myocardial tissue swelling (red arrows), cell degeneration (green arrows), and inflammatory infiltrates (black arrows) in the ePTFE and sham groups; Masson's trichrome staining exhibited fibrosis in myocardial tissues (blue staining with yellow arrows). Scale bar = 50 *μ*m. (b) The degree of fibrosis in individual groups of rabbits. ^∗^*P* < 0.05.

To understand the molecular pathogenesis of ePTFE graft-related myocardial damages, rabbits were subjected to a sham surgery (sham group) or an ePTFE implantation (ePTFE group) in the right ventricular outflow tract of the heart. A group of healthy rabbits served as the control (control group). One month after the surgery, their graft-related myocardial tissues were dissected and their sections were stained with H&E and Masson's trichrome for histological examination. As shown in Figure 1(a), while there were healthy myocardial cells in the control rabbits, there were many myocardial cells undergoing atrophy and degeneration, accompanied by some degrees of inflammatory infiltrates in the myocardial tissues from the rabbits with an ePTFE implant. In contrast, there were less pathogenic signs in the myocardial tissues from the sham group of rabbits. Quantitative analyses revealed that the degrees of fibrosis in the sham group were significantly less than those in the ePTFE group, but greater than those in the control (*P* < 0.05 for both, Figure 1(b)). Hence, implantation with an ePTFE in the right ventricular outflow tract of the heart induced the implant-related myocardial injury in rabbits.

### 3.2. ePTFE Implantation Modulates the Expression Profiles of mRNAs in the Myocardial Tissues

To explore the potential molecular pathogenesis of ePTFE implant-related myocardial tissue injury, the ePTFE implant-related myocardial tissues from each group were dissected out at one month postsurgery and their RNA transcripts were analyzed by RNA-seq. We achieved 887.04 M raw reads and 864.85 M clean reads after removal of adaptor sequences and low-quality reads. The percentage of GC content varied from 48.54 to 53.97%, and *Q*30 value varied from 93.35 to 95.79% in each sample. There were more than 89.33% of the obtained clean reads in each sample mapping to the OryCun 2.0. Thus, the RNA-seq data were quite credible.

**Figure 2:**
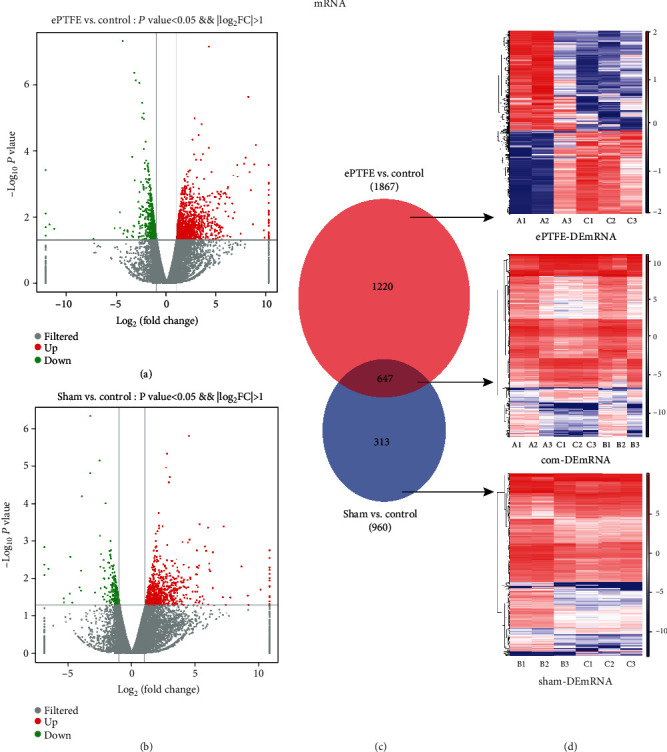
Bioinformatic analyses of DEmRNAs. The distribution of DEmRNAs in the (a) ePTFE-control comparison or the (b) sham-control comparison was analyzed by a volcano plot. Vertical lines indicate 2-fold changes in upregulation or downregulation, and the horizontal line represents *P* = 0.05; red dots refer to upregulated mRNAs, and blue points refer to downregulated mRNAs. (c) The numbers of specific and overlapped DEmRNAs from the ePTFE-control and sham-control comparisons are presented by Venn diagrams. (d) The expression patterns of the corresponding DEmRNAs are exhibited by heatmaps. Red color indicates high expression and blue indicates low expression.

In comparison with that in the control, there were 1867 DEmRNAs in the ePTFE group (ePTFE-control comparison). Among them, 1107 DEmRNAs were upregulated while 760 DEmRNAs downregulated. Similarly, there were 960 DEmRNAs in the sham group (sham-control comparison), including 685 upregulated and 275 downregulated DEmRNAs. The volcano plot revealed that these DEmRNAs were clearly separated and contained several DEmRNAs with high fold changes and strongly statistical significance (Figures 2(a) and 2(b)). Comparison of the ePTFE-control with the sham-control indicated that 647 DEmRNAs were overlapped and the remaining 1220 and 313 DEmRNAs were specific for the ePTFE-control and sham-control analysis, respectively (Figure 2(c)). The expression patterns of specific and shared DEmRNAs are shown in a heatmap (Figure 2(d)). Of interest, the most upregulated and downregulated mRNAs in the ePTFE group were LOC100009136 and BMP10, with a fold change of 935.0357 and 0.0002, respectively. Collectively, such data indicated that an ePTFE implant in the right ventricular outflow tract of the heart for 30 days modulated the expression profiles of many genes, contributing to the ePTFE implant-related myocardial injury in rabbits.

### 3.3. ePTFE Implantation Modulates the Expression Profiles of lncRNAs

**Figure 3:**
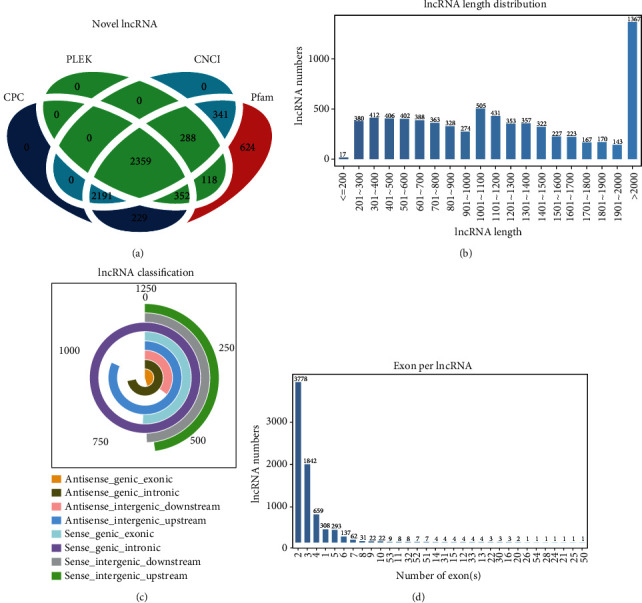
The features of lncRNAs. (a) Venn diagrams displayed lncRNA transcripts from CPC, CNCI, Pfam, and PLEK. (b) The numbers of different types of lncRNAs. (c) The length distribution of lncRNAs. (d) The number of exons per lncRNA.

Analysis of lncRNAs identified 4876 known and 2359 novel lncRNA transcripts in three groups. The novel transcripts were identified by filtering through CPC, CNCI, Pfam, and PLEK analyses (Figure 3(a)). There were 7219 lncRNAs sized ≥200 bp (Figure 3(b)). According to their direction, type, and location, these lncRNAs were classified into 8 types, of which sense genic_intronic lncRNAs were the most common (1258), followed by anti-sense_intergenic_upstream lncRNAs (1028), anti-sense_genic_intronic lncRNAs (905), sense_genic_exonic lncRNAs (638), sense_intergenic_downstream lncRNAs (623), sense_intergenic_upstream lncRNAs (601), anti-sense_genic_exonic lncRNAs (571), and anti-sense_intergenic_downstream lncRNAs (443) (Figure 3(c)). Approximately, 52.2% of lncRNAs were over two exons, and 25.5% of lncRNAs covered three exons (Figure 3(d)).

**Figure 4:**
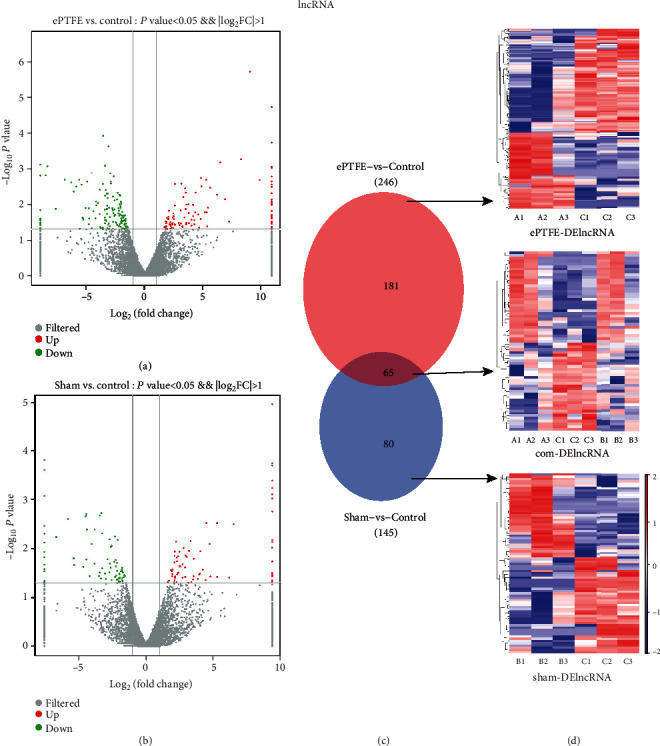
Bioinformatic analysis of DElncRNAs. The distribution of DElncRNAs in the (a) ePTFE-control comparison or the (b) sham-control comparison was analyzed by a volcano plot. Vertical lines indicate 2-fold changes in upregulation or downregulation, and the horizontal line represents *P* = 0.05; red dots refer to upregulated mRNAs, and blue points refer to downregulated mRNAs. (c) The numbers of specific and overlapped DElncRNAs from the ePTFE-control and sham-control comparisons are presented by Venn diagrams. (d) The expression patterns of the corresponding DElncRNAs are exhibited by heatmaps. Red color indicates high expression and blue indicates low expression.

**Figure 5:**
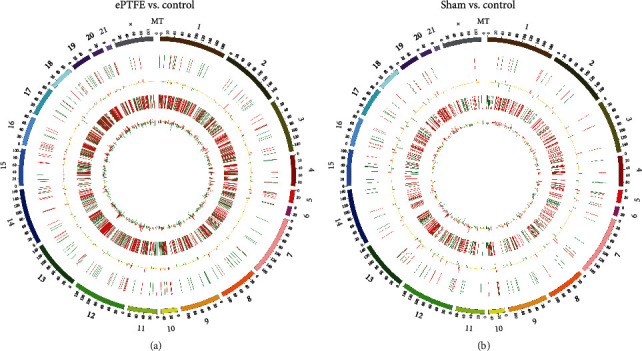
Circos plot displays the distribution of DEmRNAs and DElncRNAs on chromosomes. The distribution of DEmRNAs and DElncRNAs from the (a) ePTFE-control and (b) sham-control comparisons is exhibited by the Circos plot. The outermost circle is the distribution diagram of chromosomes. The second circle is the distribution of DElncRNAs on chromosomes, and the third circle is the histogram of DElncRNAs at different positions. The fourth circle is the distribution of DEmRNAs on chromosomes, and the innermost circle is the histogram of DEmRNAs at different positions. The higher the column, the higher the number of different genes. The red and green indicate the upregulated and downregulated genes, respectively.

In comparison with that in the control, there were 391 DElncRNAs, of which, 246 DElncRNAs are from the ePTFE-control comparison, including 110 upregulated and 136 downregulated DElncRNAs, while 145 DElncRNAs are from the sham-control comparison, including 70 upregulated and 75 downregulated DElncRNAs. The volcano plot also revealed that these DElncRNAs were clearly separated and contained several DElncRNAs with high fold changes and strongly statistical significance (Figures 4(a) and 4(b)). Comparison of the ePTFE-control with the sham-control indicated that 65 DElncRNAs were overlapped and the remaining 181 and 80 DElncRNAs were specific for the ePTFE-control and sham-control analysis, respectively (Figure 4(c)). The expression patterns of specific and shared DElncRNAs are shown in the heatmap (Figure 4(d)). In addition, the most upregulated DElncRNA was TCONS_00041400 with a fold change of 1900.7034, and the most downregulated DElncRNA was TCONS_00006875 with a fold change of 0.0028 in the ePTFE group. Further analyses uncovered that these DEmRNAs and DElncRNAs were widely distributed in different chromosomes (Figure 5). Such results indicated that an ePTFE implantation in the right ventricular outflow tract of the heart significantly altered the lncRNA expression profiles in the implant-related myocardial tissues, contributing to myocardial tissue injury in rabbits.

### 3.4. Validation of Selected DEmRNAs and DElncRNAs by Quantitative RT-PCR

**Figure 6:**
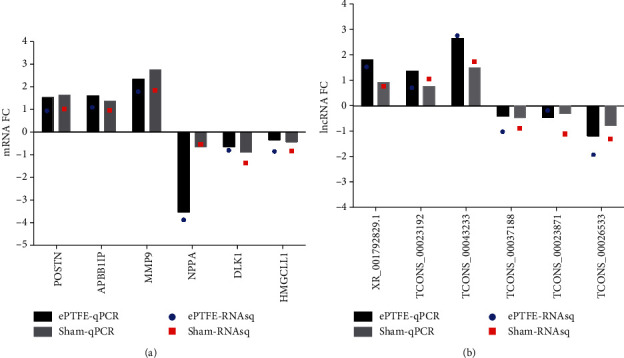
Quantitative RT-PCR validates the altered levels of some DEmRNAs and lncRNAs. The expression levels of selected (a) DEmRNAs and (b) DElncRNAs in myocardial tissues form the ePTFE and sham groups were validated by RT-qPCR. Their expression levels were compared with the data from RNA-seq.

To validate the reliability of sequencing data, 6 DEmRNAs (3 upregulated and 3 downregulated) and 6 DElncRNAs (3 upregulated and 3 downregulated) were selected and their expression was quantified by RT-PCR. In comparison with the results from RNA-seq, these DEmRNAs and DElncRNAs displayed a similar trend of altered expression (Figure 6). Such data indicated the reliability of RNA-seq data.

### 3.5. Functional Analysis of DEmRNAs Caused by ePTFE Implantation

**Figure 7:**
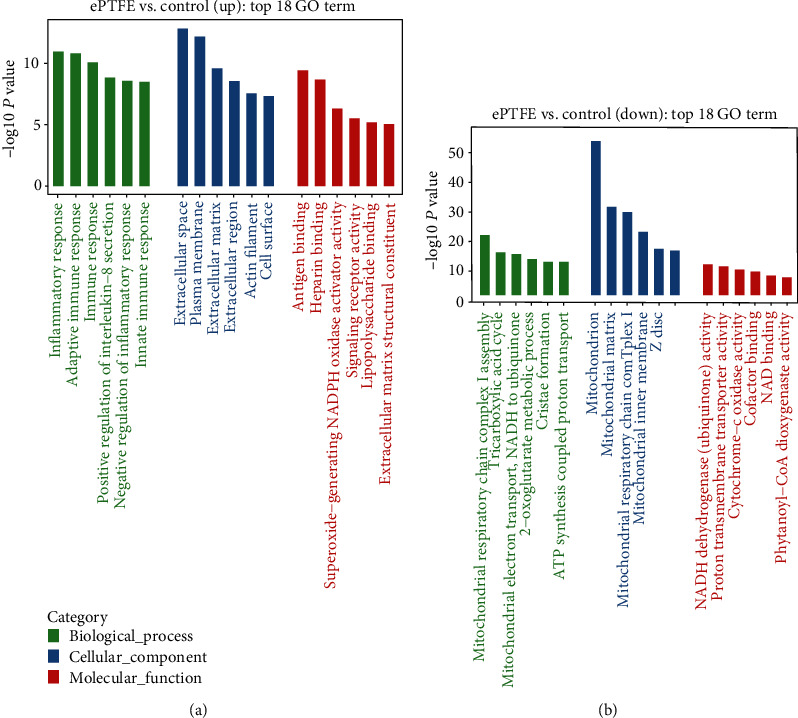
GO enrichment analysis of DEmRNAs in the ePTFE-control comparison. (a) The upregulated DEmRNAs. (b) The downregulated DEmRNAs. Data are the top 6 terms (according to *P* value) in the GO terms of BP, CC, and MF.

GO enrichment and KEGG enrichment analysis predicted the potential functions of the upregulated and downregulated DEmRNAs unique in the ePTFE-control comparison. The details of top 6 items in each GO term are displayed in Figure 7. The representative upregulated and downregulated DEmRNAs in the top 1 BP, CC, and MF terms are shown in Table 2.

**Figure 8:**
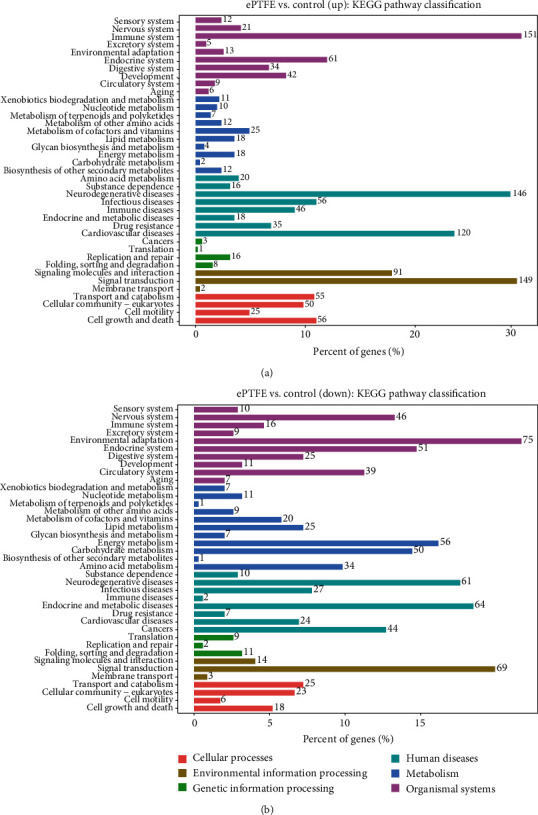
KEGG level 2 analysis of DEmRNAs in the ePTFE-control comparison. (a) The upregulated DEmRNAs. (b) The downregulated DEmRNAs.

**Figure 9:**
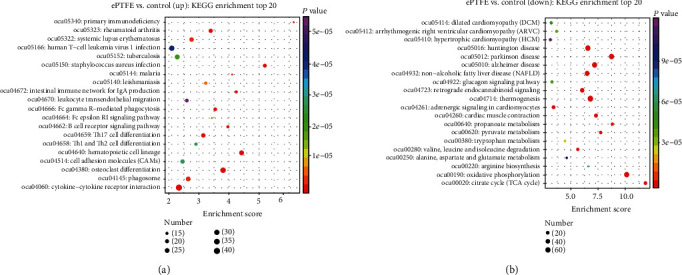
KEGG level 3 analysis of DEmRNAs in the ePTFE-control comparison. (a) The upregulated DEmRNAs. (b) The downregulated DEmRNAs. Data are presented as the sized bubbles as the number of enriched mRNAs in the top 20 pathways. The colors indicate variable *P* values and positions indicate enrichment factors.

**Figure 10:**
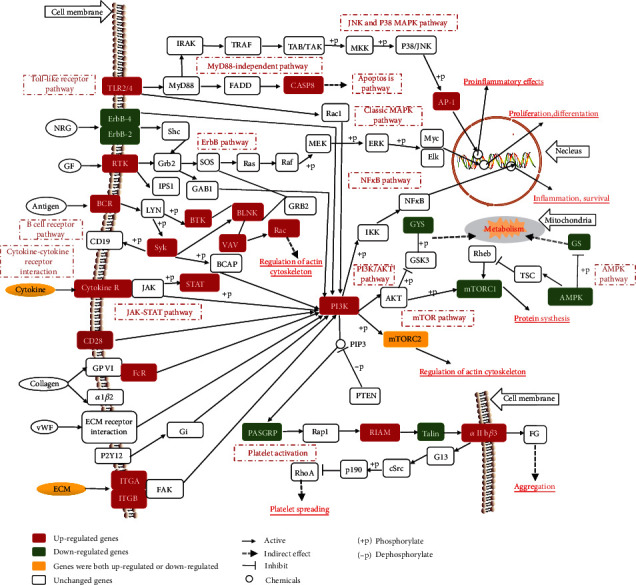
A diagram illustrates the key molecular signal pathways affected by ePTFE implantation. Bold lines indicate direct interaction, and dotted lines indicate indirect interaction. The upregulated genes are highlighted in red while the downregulated genes are shown in green. Yellow indicates both upregulated and downregulated genes.

The KEGG level 2 analysis indicated that most upregulated DEmRNAs were enriched in the immune system, signal transduction, and infectious diseases (Figure 8(a)), and the most downregulated DEmRNAs were enriched in environmental adaption, signal transduction, and endocrine and metabolic diseases (Figure 8(b)). The upregulated and downregulated DEmRNAs enriched in the top 20 KEGG pathway are shown in Figure 9. The upregulated DEmRNAs were primarily enriched in the cytokine-cytokine receptor interaction and osteoclast differentiation pathways. The downregulated DEmRNAs were mainly enriched in the oxidative phosphorylation, thermogenesis, and dilated cardiomyopathy pathways. Such data suggest that implanted ePTFE may promote immune response and extracellular matrix change, negatively affecting cell metabolism and myocardial activity. We constructed one of the important signal pathway maps to illustrate the relationship between different pathways (Figure 10).

After analyzing the function of DEmRNAs specific in ePTFE-control or sham-control comparison and common in these two comparisons, further analyses revealed that first, although both an ePTFE implantation and sham surgery caused inflammatory and immune responses, these responses caused by the ePTFE implantation were much severer than those caused by sham surgery. Secondly, many genes related to adaptive immune response changed a lot after ePTFE implantation, but there were almost no genes related to adaptive immune response changed after sham surgery. Thirdly, both an ePTFE implantation and sham surgery promoted extracellular matrix modification in myocardial tissues, but the molecular changes between two groups were different. Finally, both an ePTFE implantation and sham surgery negatively affected cell metabolism and cardiac muscular contraction, particularly for the ePTFE implantation.

### 3.6. Coexpression of DEmRNAs and DElncRNAs Caused by ePTFE Implantation

To understand the relationship between the DEmRNAs and DElncRNAs, we analyzed their coexpression. We found that 221001 pairs of DElncRNAs and DEmRNAs were coexpressed, according to the criterion ∣PCC | >0.8 and *P* < 0.05. The top 10 DElncRNAs, such as TCONS_00043233, were mainly enriched in the metabolism-related GO term, including the mitochondrial respiratory chain complex I assembly, inflammatory response, immune response, and vascular smooth muscle cell development. Those top 10 DElncRNAs were also enriched in the oxidative phosphorylation, osteoclast differentiation, and cardiac muscular contraction pathways. Hence, the functions of DElncRNAs were highly similar to those of DEmRNAs.

### 3.7. *cis*- and *trans*-Regulation of DElncRNAs Caused by ePTFE Implantation

**Figure 11:**
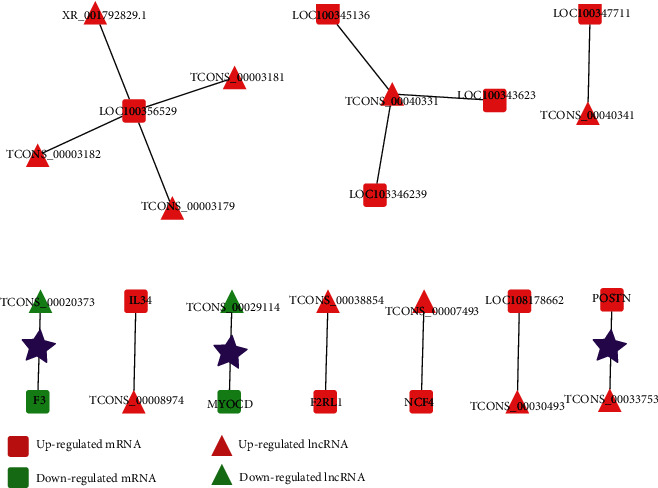
lncRNA-mRNA *cis*-regulation network. The lncRNA-mRNA *cis*-regulation networks were established based on the GO term of immune response and extracellular matrix (marked with black star).

**Figure 12:**
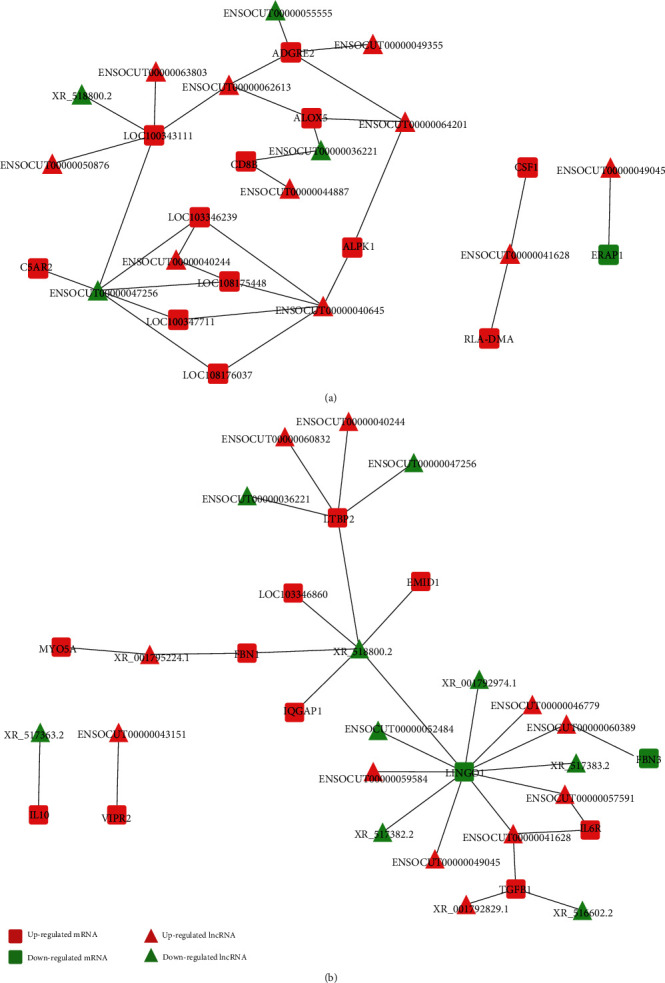
lncRNA-mRNA *trans*-regulation network. The lncRNA-mRNA *trans*-regulation networks were established based on the GO term of (a) immune response and (b) extracellular matrix.

According to the specific GO term, there were 117 DEmRNAs in the immune response term and 52 DEmRNAs in the smooth muscle cell and extracellular matrix terms. There were 12 pairs of DElncRNAs and DEmRNAs involved in the *cis*-regulation on the expression of immune response-related genes and 3 pairs of them in the *cis*-regulation on the expression of smooth muscle cell and extracellular matrix-related genes (Figure 11). However, there were many DElncRNAs involved in the *trans*-regulation on DEmRNAs in the immune response term and smooth muscle cell term and extracellular matrix terms, and the top 30 in each part were used for construction of a *trans*-regulation network (Figure 12).

## 4. Discussion

Implantation with ePTFE grafts or ePTFE membranes is a common procedure for the reconstruction of blood flow and cardiac valves [1, 7], but it can cause adverse reactions in the relevant tissues, impairing their function [6, 9, 28]. Extensive researchers have made great efforts to improve the properties of existing materials by changing the surface tomography and adding drug and antibody coating [29, 30]. However, there are few effective approaches to clinical application [14].

Although many works have been done to reveal the mechanisms underlying the ePTFE-related complications, the molecular pathogenesis of adverse reactions caused by ePTFE implantation currently remains unclear. In addition, there is no information on the pathological changes and transcriptome profile alternations in the ePTFE-related myocardial tissues and no available knowledge about the function and regulatory network of ePTFE-related mRNAs and lncRNAs [23–25]. In this study, we employed a rabbit model of ePTFE implantation in the right ventricular outflow tract of the heart. We observed that ePTFE implantation for 30 days caused the relevant myocardial cell degeneration and atrophy, myocardial tissue inflammation, and fibrosis in rabbits, similar to that in the ePTFE-implanted vessels [9, 12]. The high degrees of fibrosis in the myocardial tissues of the ePTFE-implanted rabbits may cause local flow obstruction and abnormal myocardial contractility, mimicking clinical observations in patients with ePTFE implants [2, 31].

We identified 1867 ePTFE-related DEmRNAs in myocardial tissues of rabbits, which were twice more than in the sham group. In addition, only 35% of DEmRNAs in the ePTFE group shared with that in the sham group, and the relative levels of DEmRNAs in the ePTFE group were higher than those in the sham group. The greater numbers of DEmRNAs and higher levels of their transcripts indicated that ePTFE implant altered the transcriptome profiles in myocardial tissues. Furthermore, functional prediction revealed that the upregulated DEmRNAs were mainly involved in immune and inflammatory responses and extracellular matrix remodeling processes while the downregulated DEmRNAs were mostly enriched in metabolism and cardiac remodeling. Hence, ePTFE implant caused inflammation and immune responses and inhibited cardiac cell metabolism, which would impair the heart function.

lncRNAs, a kind of noncoding RNAs with transcripts longer than 200 nucleotides, can regulate the expression of mRNA and are potential therapeutic targets [32]. lncRNAs can also be crucial regulators of pathological processes, including tumor [33], cardiovascular diseases [34, 35], inflammatory diseases [36], and vascular injury-related neointimal hyperplasia [37]. However, there is little information on the role of lncRNAs in adverse reaction caused by artificial materials. In this study, we identified 2359 novel lncRNAs and 246 DElncRNAs in the myocardial tissues from the ePTFE group. Furthermore, we found that some DElncRNAs were coexpressed with some DEmRNAs, mainly through the *trans*-regulation, leading to a regulatory network. Thus, these DElncRNAs may be potential new molecular targets to reduce the ePTFE-related complications.

Previous studies have suggested material implantation can cause a FBR, which is mainly involved in inflammatory stimuli, immune response, endothelial cell damages, vascular smooth muscle cells (VSMCs) switching from a quiescent contractile phenotype to an active synthetic mode, and cardiac injury [38, 39]. Our present studies have confirmed some mechanism underlying the FBR found by others [40–43]. In this study, we found that ePTFE implantation upregulated MAPK15 (fold change 8.59) and MAPK8IP1 (fold change 2.66) mRNA transcripts in myocardial tissues of rabbits, supporting the notion that vascular smooth muscular MAPK14 is required for neointimal hyperplasia by activating the MAPK pathway [43]. Similarly, our RNA-seq in myocardial tissue shared some similarity with the molecular changes in vessel tissues [24, 25]. For example, we found that ePTFE implant increased ACTG2 and MYOM1 mRNA transcripts, mimicking the enhanced MMP13 and COL11A1 expression in the osteo/chondrogenic pathways in porcine vessel tissues [24]. These indicated that ePTFE implantation changed some mRNA transcripts related to matrix accumulation in different cardiovascular tissues in different species of animals. We are interested in further investigating whether similar lncRNA transcriptome profiles change during the process of FBR in different tissues and whether different biomedical material implants cause FBR with similar molecular mechanisms.

Our findings not only confirmed the existing mechanisms involved in the process of FBR but also provided novel potential key mechanisms. These findings may help in uncovering new therapeutic targets for prevention and intervention of ePTFE-related complications [30]. For example, we found that both innate and adaptive immune responses might be involved in the ePTFE-related adverse reactions, which extended previous observations that only innate immune responses existed in FBR caused by artificial material implantation [17, 19, 44, 45]. Many molecules and signal pathways were involved in the mechanisms underlying the ePTFE-related adverse reactions, and they might interact with each other in multiple ways, which increased the difficulty of learning the underlying mechanisms. However, the signal pathway map, based on our sequencing data, can be constructed to help us learn the relationship between different molecules and pathways. According to the signal pathway map (Figure 7), the downstream of many inflammatory pathways is the PI3K/AKT pathway, which is also the upstream of cell proliferation or extracellular matrix-related pathways. Furthermore, the PI3K/AKT pathway is also involved in the platelet activation pathway. Therefore, the PI3K/AKT signal pathway may be a new target to reduce platelet activation and related adverse reaction caused by ePTFE implantation.

Recently, there are many research approaches to modification of targeted genes and application of synthetic materials to reduce implant-related complications [5, 46–48]. However, most of the gene-targeting strategies are not based on implant-related mechanisms. Our findings predicted the potential molecular mechanisms underlying the ePTFE-related adverse reactions, which should help in targeting specific genes to achieve therapeutic efficacy. Given that implanting different biomaterials in different organs may cause varying adverse reactions with varying mechanism, it is important to design an optimal animal model to explore the potential molecular mechanisms underlying the specific implant-related adverse reaction. Our findings may provide a new and easier animal model to study the cardiac tissue injury induced by a specific implant without complex cardiopulmonary bypass.

## 5. Conclusions

Transcriptome profile alternation was different between ePTFE implantation and surgery in the heart without material implantation, which indicated that the mechanism of adverse reaction caused by ePTFE implantation was unique. Hence, our findings may uncover novel potential targets to improve the properties of medical materials and new solutions to ameliorate the postoperation complications caused by an ePTFE implantation.

## Figures and Tables

**Table 1:** The sequences of primers for RT-PCR analysis.

Gene	Forward primer (5′-3′)	Reverse primer (5′-3′)
GAPDH	GACTTCAACAGTGCCACC	TGCTGTAGCCAAATTCGT
POSTN	CCACTACAACACAGCGTTAT	TCCAAGTTGTCCCAAGCC
APBB1IP	CTAAATGAGTCCTTAAACGCAC	TCTGCTCAGCTTCACTTATGT
MMP9	CGTCTTCCAGTACCGAGAG	CACCTGGTCCACTAGGTT
NPPA	GTGAGCTTCCTCTTCTGTCT	GATCTGCGTTGGACATGG
DLK1	TTGCTCCTGCTGGCTTTC	ACCTGCACACATTGTCATC
HMGCLL1	GGATTAGGAGGTTGCCCTTAT	GATTCACACCTGTATTGAGCC
XR_001792829.1	ATCTCGACCACTTCTCGG	CGACATCTACGTTCTTGGTCA
TCONS_00023192	GAGCCCAAGGCAGTGTTA	TTGACTGCACTCTGCTAGAC
TCONS_00043233	GGTAGCACATACTACGCGA	GTCAGACTGTTCAGTTGTAGG
TCONS_00037188	CCACACACTGTGCAAATAAT	GTCTGGTTTCTGGTTGAGATAC
TCONS_00023871	GGCTGATCTGGCTGGCTA	GGATGGTCGTCCTCTTCG
TCONS_00026533	TTAGCCGGAGCTTGGAACA	AGGTTATGAGGCTCCCAC

**Table 2:** The representative DEmRNAs in the top GO terms.

GO term	Category	Regulation station	Representative DEmRNAs
Inflammatory response	Biological process	Up	CCL2, CD180, CD44, CHI3L1, CXCL8, IL1RAP, IL34, TLR2
Extracellular space	Cellular component	Up	ADAMTS15, COL11A1, DKKL1, MMP14, NAPSA, NPC2, OAS3, TIMP1
Antigen binding	Molecular function	Up	CD48, LAG3, TGFB1
Mitochondrial respiratory chain complex I assembly	Biological process	Down	ACAD9, NDUFA11, NDUFB1, NDUFAF7, NDUFC1, NDUFS4, NDUFS5, OXA1L
Mitochondrion	Cellular component	Down	ATP5H, COQ7, CPS1, DLAT, OXCT1, TIMM44, TRAK2, VDAC2
NADH dehydrogenase (ubiquinone) activity	Molecular function	Down	NDUFA12, NDUFA5, NDUFA7, NDUFB8, NDUFC1, NDUFC2, NDUFS1, NDUFS3

## Data Availability

Our RNA sequencing (RNA-seq) data were submitted to the SRA database of NCBI (SRP274056, https://pubmed.ncbi.nlm.nih.gov/sra). Other datasets used and analyzed during the current study are available from the corresponding author upon reasonable request.

## Abbreviations

ePTFE: Expanded polytetrafluoroethylene
BT: Blalock-Taussig
FBR: Foreign body reaction
lncRNA: Long noncoding RNA
NCBI: National Center for Biotechnology Information
RIN: RNA integrity number
RNA-seq: RNA sequence
OryCun: *Oryctolagus cuniculus* reference genome
FPKM: Fragments per kilobase of exon model per million reads mapped
FC: Fold change
DEmRNAs: Differentially expressed mRNAs
DElncRNAs: Differentially expressed lncRNAs
GO: Gene Ontology
KEGG: Kyoto Encyclopedia of Genes and Genomes
BP: Biological process
CC: Cellular component
MF: Molecular function
PCC: Pearson correlation coefficient.

## References

[1] Shibutani S., Obara H., Matsubara K. (2020). Midterm results of a Japanese prospective multicenter registry of heparin-bonded expanded polytetrafluoroethylene grafts for above-the-knee femoropopliteal bypass. *Circulation Journal*.

[2] Ando M., Takahashi Y. (2009). Ten-year experience with handmade trileaflet polytetrafluoroethylene valved conduit used for pulmonary reconstruction. *The Journal of Thoracic and Cardiovascular Surgery*.

[3] Sasikumar N., Hermuzi A., Fan C. S. (2017). Outcomes of Blalock-Taussig shunts in current era: a single center experience. *Congenital Heart Disease*.

[4] Ito T., Maekawa A., Yamana K., Yoshizumi T., Sunada M. (2010). Use of an expanded polytetrafluoroethylene patch as an artificial leaflet in mitral valve plasty: an early experience. *The Annals of Thoracic Surgery*.

[5] Preis M., Schneiderman J., Koren B. (2016). Co-expression of fibulin-5 and VEGF165 increases long-term patency of synthetic vascular grafts seeded with autologous endothelial cells. *Gene Therapy*.

[6] Tzchori I., Falah M., Shteynberg D. (2018). Improved patency of ePTFE grafts as a hemodialysis access site by seeding autologous endothelial cells expressing fibulin-5 and VEGF. *Molecular Therapy*.

[7] Ambler G. K., Twine C. P. (2018). Graft type for femoro-popliteal bypass surgery. *Cochrane Database of Systematic Reviews*.

[8] Liu Y., Munisso M. C., Mahara A. (2018). A surface graft polymerization process on chemically stable medical ePTFE for suppressing platelet adhesion and activation. *Biomaterials Science*.

[9] Kirkton R. D., Prichard H. L., Santiago-Maysonet M., Niklason L. E., Lawson J. H., Dahl S. L. M. (2018). Susceptibility of ePTFE vascular grafts and bioengineered human acellular vessels to infection. *The Journal of Surgical Research*.

[10] Lei Z. Y., Li J., Liu T., Shi X. H., Fan D. L. (2019). Autologous vascularization: a method to enhance the antibacterial adhesion properties of ePTFE. *The Journal of Surgical Research*.

[11] Shan Y., Jia B., Ye M., Shen H., Chen W., Zhang H. (2018). Application of heparin/collagen-REDV selective active interface on ePTFE films to enhance endothelialization and anticoagulation. *Artificial Organs*.

[12] Lu S., Zhang P., Sun X. (2013). Synthetic ePTFE grafts coated with an anti-CD133 antibody-functionalized heparin/collagen multilayer with rapid in vivo endothelialization properties. *ACS Applied Materials & Interfaces*.

[13] Chen L., He H., Wang M., Li X., Yin H. (2017). Surface coating of polytetrafluoroethylene with extracellular matrix and anti-CD34 antibodies facilitates endothelialization and inhibits platelet adhesion under sheer stress. *Tissue Eng Regen Med.*.

[14] Neděla O., Slepička P., Švorčík V. (2017). Surface modification of polymer substrates for biomedical applications. *Materials (Basel)*.

[15] Bastijanic J. M., Kligman F. L., Marchant R. E., Kottke-Marchant K. (2016). Dual biofunctional polymer modifications to address endothelialization and smooth muscle cell integration of ePTFE vascular grafts. *Journal of Biomedical Materials Research. Part A*.

[16] Gregory E. K., Webb A., Vercammen J. M. (2018). Inhibiting intimal hyperplasia in prosthetic vascular grafts via immobilized all-trans retinoic acid. *Journal of Controlled Release*.

[17] Jansen L. E., Amer L. D., Chen E. Y. (2018). Zwitterionic PEG-PC hydrogels modulate the foreign body response in a modulus-dependent manner. *Biomacromolecules*.

[18] Gu B., Papadimitrakopoulos F., Burgess D. J. (2018). PLGA microsphere/PVA hydrogel coatings suppress the foreign body reaction for 6 months. *Journal of Controlled Release*.

[19] Lee J. S., Shin B. H., Yoo B. Y. (2019). Modulation of foreign body reaction against PDMS implant by grafting topographically different poly(acrylic acid) micropatterns. *Macromolecular Bioscience*.

[20] Pang J. H., Farhatnia Y., Godarzi F. (2015). In situ endothelialization: bioengineering considerations to translation. *Small*.

[21] Bose S., Robertson S. F., Bandyopadhyay A. (2018). Surface modification of biomaterials and biomedical devices using additive manufacturing. *Acta Biomaterialia*.

[22] Yamamoto Y., Yamagishi M., Maeda Y. (2020). Histopathologic analysis of explanted polytetrafluoroethylene-valved pulmonary conduits. *Seminars in Thoracic and Cardiovascular Surgery*.

[23] Hamdan A. D., Aiello L. P., Quist W. C. (1995). Isolation of genes differentially expressed at the downstream anastomosis of prosthetic arterial grafts with use of mRNA differential display. *Journal of Vascular Surgery*.

[24] Kwon S. H., Li L., Terry C. M. (2018). Differential gene expression patterns in vein regions susceptible versus resistant to neointimal hyperplasia. *Physiological Genomics*.

[25] Willis D. J., Kalish J. A., Li C. (2004). Temporal gene expression following prosthetic arterial grafting. *The Journal of Surgical Research*.

[26] Bolger A. M., Lohse M., Usadel B. (2014). Trimmomatic: a flexible trimmer for Illumina sequence data. *Bioinformatics*.

[27] Kim D., Langmead B., Salzberg S. L. (2015). HISAT: a fast spliced aligner with low memory requirements. *Nature Methods*.

[28] Lucereau B., Bellissard A., Beck F. (2019). Non-anastomotic complete ePTFE axillobifemoral bypass disruption and thrombosis following shoulder dislocation. *EJVES Short Rep.*.

[29] Nemani S. K., Annavarapu R. K., Mohammadian B. (2018). Surface modification of polymers: methods and applications. *Advanced Materials Interfaces*.

[30] Oliva N., Unterman S., Zhang Y., Conde J., Song H. S., Artzi N. (2015). Personalizing biomaterials for precision nanomedicine considering the local tissue microenvironment. *Advanced Healthcare Materials*.

[31] Munarin F., Kant R. J., Rupert C. E., Khoo A., Coulombe K. L. K. (2020). Engineered human myocardium with local release of angiogenic proteins improves vascularization and cardiac function in injured rat hearts. *Biomaterials*.

[32] Kumar M. M., Goyal R. (2017). LncRNA as a therapeutic target for angiogenesis. *Current Topics in Medicinal Chemistry*.

[33] Peng W. X., Koirala P., Mo Y. Y. (2017). LncRNA-mediated regulation of cell signaling in cancer. *Oncogene*.

[34] Chen Y., Li X., Li B. (2019). Long non-coding RNA ECRAR triggers post-natal myocardial regeneration by activating ERK1/2 signaling. *Molecular Therapy*.

[35] Wang L., Zhang J. (2020). Exosomal lncRNA AK139128 derived from hypoxic cardiomyocytes promotes apoptosis and inhibits cell proliferation in cardiac fibroblasts. *International Journal of Nanomedicine*.

[36] Zhao L., Xia M., Wang K. (2020). A long non-coding RNA IVRPIE promotes host antiviral immune responses through regulating interferon *β*1 and ISG expression. *Frontiers in Microbiology*.

[37] Zhang C. J., Liu C., Wang Y. X. (2019). Long non-coding RNA-SRA promotes neointimal hyperplasia and vascular smooth muscle cells proliferation via MEK-ERK-CREB pathway. *Vascular Pharmacology*.

[38] Feng L., Ning R., Liu J. (2020). Silica nanoparticles induce JNK-mediated inflammation and myocardial contractile dysfunction. *Journal of Hazardous Materials*.

[39] Wu P., Zhang Z., Ma G., Li J., Zhou W. (2020). Transcriptomics and metabolomics reveal the cardioprotective effect of compound Danshen tablet on isoproterenol-induced myocardial injury in high-fat-diet fed mice. *Journal of Ethnopharmacology*.

[40] Sun L. J., Qiao W., Xiao Y. J., Cui L., Wang X., Ren W. D. (2019). Naringin mitigates myocardial strain and the inflammatory response in sepsis-induced myocardial dysfunction through regulation of PI3K/AKT/NF-*κ*B pathway. *International Immunopharmacology*.

[41] Fang H., Yang S., Luo Y. (2018). Notoginsenoside R1 inhibits vascular smooth muscle cell proliferation, migration and neointimal hyperplasia through PI3K/Akt signaling. *Scientific Reports*.

[42] Jain M., Dhanesha N., Doddapattar P. (2020). Smooth muscle cell-specific fibronectin-EDA mediates phenotypic switching and neointimal hyperplasia. *The Journal of Clinical Investigation*.

[43] Wu W., Zhang W., Choi M. (2019). Vascular smooth muscle-MAPK14 is required for neointimal hyperplasia by suppressing VSMC differentiation and inducing proliferation and inflammation. *Redox Biology*.

[44] Lucke S., Walschus U., Hoene A. (2018). The in vivo inflammatory and foreign body giant cell response against different poly(l-lactide-co-d/l-lactide) implants is primarily determined by material morphology rather than surface chemistry. *Journal of Biomedical Materials Research. Part A*.

[45] Klopfleisch R., Jung F. (2017). The pathology of the foreign body reaction against biomaterials. *Journal of Biomedical Materials Research. Part A*.

[46] Yu X., Wang H., Shao H., Zhang C., Ju X., Yang J. (2020). PolyI:C upregulated CCR5 and promoted THP-1-derived macrophage chemotaxis via TLR3/JMJD1A signalling. *Cell Journal*.

[47] Yang N., Tan R. P., Chan A. H. P. (2020). Immobilized macrophage colony-stimulating factor (M-CSF) regulates the foreign body response to implanted materials. *ACS Biomaterials Science & Engineering*.

[48] Huynh C., Shih T. Y., Mammoo A. (2019). Delivery of targeted gene therapies using a hybrid cryogel-coated prosthetic vascular graft. *PeerJ*.

